# Allelic Variation in a Willow Warbler Genomic Region Is Associated with Climate Clines

**DOI:** 10.1371/journal.pone.0095252

**Published:** 2014-05-01

**Authors:** Keith W. Larson, Miriam Liedvogel, BriAnne Addison, Oddmund Kleven, Terje Laskemoen, Jan T. Lifjeld, Max Lundberg, Susanne Åkesson, Staffan Bensch

**Affiliations:** 1 Department of Biology, Centre for Animal Movement Research, Ecology Building, Lund University, Lund, Sweden; 2 School of Science and Health, University of Western Sydney, Penrith, New South Wales, Australia; 3 Natural History Museum, University of Oslo, Oslo, Norway; 4 Norweigen Institute of Nature Research, Oslo, Norway; BiK-F Biodiversity and Climate Research Center, Germany

## Abstract

Local adaptation is an important process contributing to population differentiation which can occur in continuous or isolated populations connected by various amounts of gene flow. The willow warbler (*Phylloscopus trochilus*) is one of the most common songbirds in Fennoscandia. It has a continuous breeding distribution where it is found in all forested habitats from sea level to the tree line and therefore constitutes an ideal species for the study of locally adapted genes associated with environmental gradients. Previous studies in this species identified a genetic marker (AFLP-WW1) that showed a steep north-south cline in central Sweden with one allele associated with coastal lowland habitats and the other with mountainous habitats. It was further demonstrated that this marker is embedded in a highly differentiated chromosome region that spans several megabases. In the present study, we sampled 2,355 individuals at 128 sites across all of Fennoscandia to study the geographic and climatic variables associated with the allele frequency distributions of WW1. Our results demonstrate that 1) allele frequency patterns significantly differ between mountain and lowland populations, 2) these allele differences coincide with extreme temperature conditions and the short growing season in the mountains, and milder conditions in coastal areas, and 3) the northern-allele or “altitude variant” of WW1 occurs in willow warblers that occupy mountainous habitat regardless of subspecies. Finally these results suggest that climate may exert selection on the genomic region associated with these alleles and would allow us to develop testable predictions for the distribution of the genetic marker based on climate change scenarios.

## Introduction

The early stages of speciation often begin with divergent selection for locally adapted traits. This can occur in continuously distributed populations connected by gene flow [1], [2] although it is probably more prevailing in isolated allopatric populations [3], [4]. The degree of divergence reflects the balance between the selection for an adaptive trait, and gene flow from nearby populations [2], [3]. In the early stages of population divergence populations share most of the ancestral genetic variation. Genes underlying traits under divergent selection are expected to diverge faster, whereas changes in neutral genetic variation is a slow process in larger populations as governed by genetic drift [2], [3], [5], [6]. Hence, neutral genetic markers often do not reveal population structure in recently diverged populations because of insufficient time for drift to result in divergence or because a balance between gene flow and genetic drift tends to homogenize population differences [7].

Divergence can result from selection on adaptive traits in contrasting phenotypes. For example, divergent selection in the spider, *Agelenopsis aperta*, has resulted in both desert and riparian habitat related phenotypes [8]. In other examples, specialization on either alfalfa (*Medicago sativa*) or red clover (*Trifolium pratense*) by pea aphids (*Acyrthosiphon pisum*) has resulted in two divergent ecotypes [9], [10]. Local adaptations are not just restricted to discrete habitats since they also arise along environmental gradients that can result in a cline [11]. For example, the European common frog (*Rana temporaria*) shows clines in both morphological and reproductive traits in relation to altitude in mountain ranges across Europe [11], while in some Caribbean island bananaquit (*Coereba flaveola*) populations clines in colour polymorphisms are associated with rainfall patterns and altitude [12], [13].

Two willow warbler (*Phylloscopus trochilus*) subspecies representing distinct migratory phenotypes, or “migratypes”, meet in secondary contact and form a migratory divide in central Sweden. Across this divide, the northern *P. t. acredula* migrates south-southeast to winter from the east to south Africa [14], while the southern *P. t. trochilus*, migrates southwest to winter in West Africa [14]. Populations of the two migratypes share the same mtDNA haplotypes and alleles at 12 microsatellite loci at almost identical frequencies resulting in estimated F_st_-values very close to zero [15], [16]. In contrast, the contact zone between the two migratypes show steep clines for two phenotypic traits; morphology (mainly wing length and body mass) and feather stable nitrogen-isotopes (*δ* ^15^N), which represent a proxy for the wintering grounds [14]. Moreover, two bi-allelic amplified fragment length polymorphism (AFLP) derived genetic markers, WW1 and WW2 [14]–[17] are strongly differentiated between the migratypes (F_st_-values >0.6). While previous analysis for WW1 and WW2 showed that both allele frequency clines are steepest at the migratory divide in central Sweden and that the peak frequencies of the northern alleles reach >0.9 in the northernmost part of Scandinavia, they displayed very different clines along the eastern side of the Baltic Sea [14]–[17]. At the WW2 locus, the “northern-allele” has a high frequency (>0.9) from northern Sweden and throughout the eastern side of the Baltic Sea, in Finland and south to Lithuania [15]. In contrast, the frequency of the WW1 locus for the “northern-allele” drops in southern Finland (where we find the *acredula* migratype) to low frequencies similar to southern Sweden (where we find the *trochilus* migratype). These different cline patterns strongly suggest that the same selective processes cannot maintain variation at these loci.

The concordant cline patterns between WW2 and feather *δ* ^15^N suggest that “northern” and “southern” alleles could be associated with genes shaping the different migratory programs or represent adaptations to conditions on their different wintering grounds in Africa [15]. The selection processes governing the clines for the WW1 locus are less apparent, although we can, based on the geographic distribution of the alleles, exclude its association between the two migratypes. From earlier data we know that the northern-allele of WW1 in *acredula* populations is predominantly found in high elevation populations of the western mountains of northern Sweden [17]. As we follow the west-to-east or mountain-to-lowland altitude gradient the frequency of southern-allele at WW1 increases in *acredula*. In lowland populations within Sweden, the southern-allele is almost fixed in *trochilus* populations as it is for *acredula* populations in southern Finland where lowland habitats dominate. It is this contrast with the northern-allele found in the mountain populations of *acredula* and the substantial proportion of southern-alleles in the lowland *acredula* that suggests some form of local adaptation correlated with altitude or environmental conditions associated with these habitats.

The sequences surrounding the alleles at the WW1 locus are highly divergent with a coalescence time estimated to be several million years old [18] and must therefore have been segregating in the willow warbler population long before the species colonized northern Europe after the last glaciation. Using the zebra finch (*Taeniopygia guttata*) genome [19] as reference, WW1 has been mapped to chromosome 3. By examining 14 markers along a 7 Mb region surrounding the WW1 locus, Lundberg et al. [20] identified a 2.5 Mb chromosomal region that was highly differentiated between the subspecies (peak F_st_>0.6) and substantially above the background (F_st_<0.02). This 2.5 Mb region contains >20 protein coding genes in the zebra finch genome of which several have been shown to have genetic variation that is in strong linkage disequilibrium (LD) with the alleles at WW1 [20]. However, none of these genes have annotated functions indicating a role in climate adaptation. Although we have yet to identify the genes under divergent selection, we expect the allelic distribution of WW1 to closely follow that of the focal genes.

In this study we focus on the geographic WW1 allele frequency distribution pattern using a substantially larger data set than [17] to investigate how the northern-allele is associated with latitude, longitude and altitude. Furthermore, if the northern-allele is under positive selection for local adaption to high latitude and altitude habitats, we would predict that environmental conditions related to breeding season temperature extremes, growing season length, and productivity will best explain the observed pattern in allele frequency. Specifically, high latitude and altitude habitats are associated with shorter and delayed growing seasons and larger and more extreme climate fluctuations. This is especially so at the beginning and end of the breeding season, which provides a much shorter window for migratory birds to breed. Further, we predict the cline for WW1 allele frequencies, matching environmental parameters, to be smooth and gradual from south-to-north in Finland, where mountains are absent except in the far north on the border of Norway, reflecting gradual changes in habitats, with the extreme northern latitudes more similar to mountain habitats in Sweden and Norway. Due to the altitude gradient running perpendicular to the latitudinal gradient in the north of Sweden and Norway, we predict the clines to be more complex, narrower, and oriented east-to-west reflecting the lowland-to-mountain-to-lowland gradient across Scandinavia. Of particular interest from this expanded dataset is the addition of samples from across the entirety of Norway including mountainous sites south of the latitude associated with the migratory divide in Sweden (*trochilus* subspecies) and low elevation sites along the coast north of the migratory divide. This extensive dataset further allows us to examine whether the northern-allele is common among *trochilus* populations breeding at high altitudes and whether selection favours the southern-allele in the habitats found at low altitudes and with milder climates along the coast in Norwegian *acredula*.

## Materials and Methods

### Field and lab work

**Figure 1:**
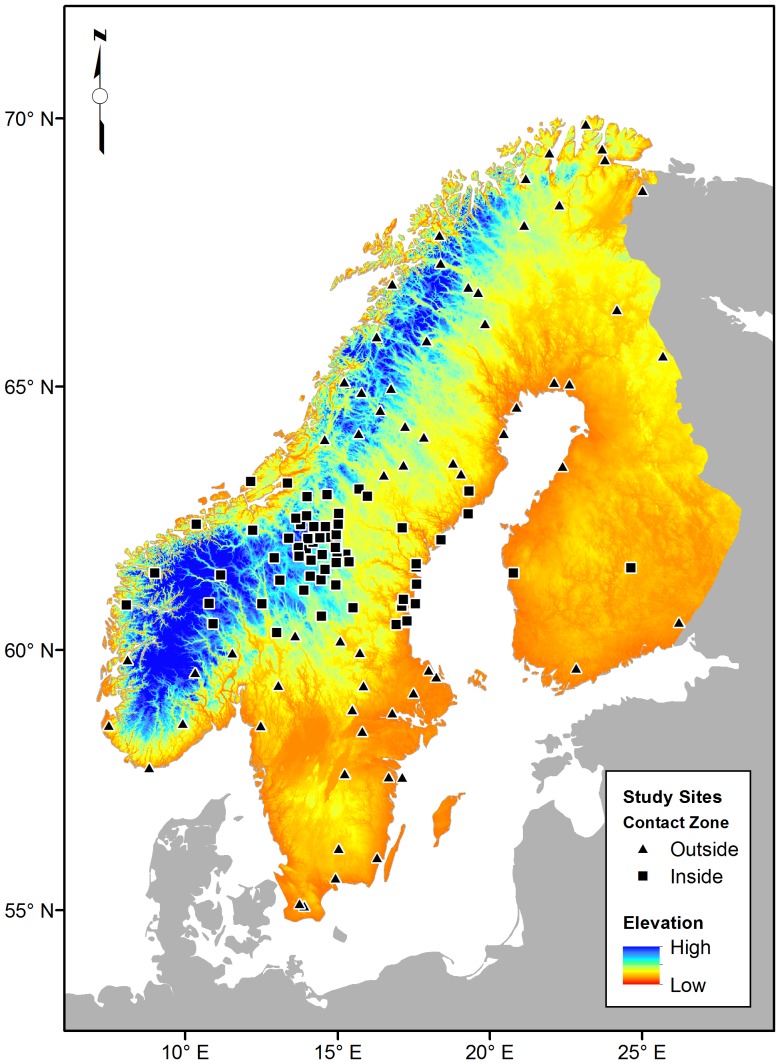
Maps of Scandinavia with backgrounds representing a digital elevation model (DEM) with Sample site locations. Sample sites within the willow warbler contact zone have square symbols, while those outside triangles. The maps have a 30 arc-second resolutions and is projected using the Swedish RT 90 0 gon Mercator projection.

Between 1996 and 2011 we collected samples at 128 sites across their breeding grounds in Fennoscandia, in Sweden (N = 85), Norway (N = 35), and Finland (N = 8) from 2,335 willow warblers (Figure 1). At each site we captured between 10 and 20 individual adult males and collected morphometric measures (e.g., wing chord, tarsus, body mass, and plumage colour), blood for DNA analysis, and the first primary flight feather for *δ* ^13^C and *δ* ^15^N analysis [14], [16], [21]. We caught all males in mist-nets with an audio-lure playback using the same willow warbler song. All blood samples were stored in DNA buffer and brought back to the lab for analysis. Procedures for extracting DNA and subsequent bi-allelic genotyping of individuals for WW1 follow previously published methods [17]. In brief, the WW1 polymorphism was identified in an AFLP genomic scan and after sequencing the fragment excised from the polyacrylamide gel, inverse PCR was used to identify the flanking sequences of the AFLP cut sites [17]. The AFLP polymorphism between the northern (presence) and southern (absence) allele was caused by a mutation in the selective primer region at the EcoRI end of the fragment. This site can been genotyped by RFLP-PCR, by using primers amplifying both alleles across this site, followed by digestion with a restriction enzyme that cuts the southern allele.

### Geographic analysis

We used the full set of sampling sites (N = 128; Figure 1) to determine the northern-allele frequency distribution for WW1 across Fennoscandia. To assess clinal variation in the northern-allele of WW1 we used a generalized additive model (GAM) to perform a logistic regression analysis. We chose GAM because it allows us to incorporate both linear and nonlinear predictors. In this model the northern-allele frequency for WW1 at each site was used as a response variable with a binomial error structure and the logit link function with the sample site geographic coordinates (i.e. latitude, longitude, and their interaction) and altitude as predictors. We tested the interaction between latitude and longitude as the Scandinavian Peninsula, and the mountains that dominate this region, is oriented northeast to southwest. Further, we predicted that the latitudinal effect will be stronger in Finland where lowlands dominate, while latitude in Scandinavia is highly correlated with altitude reflecting the northern position of the mountains, especially in Sweden. We used GAM, as implemented in the *mgcv* R package [22]. The significance of latitude, longitude, their interaction and/or altitude in this model would indicate geographic differentiation likely representing adaptions to mountain or high latitude habitats.

To visualize the cline we used the Spatial Analyst ordinary kriging function in ArcMap 10 [23] to create an interpolated continuous grid surface representing the observed northern-allele frequency distribution for the entire region. We then used the resulting interpolated surface to create allele frequency contours in 0.1 increments with the 0.5 isocline representing the center of the cline.

### Environmental analysis

Again, we used the northern-allele frequency for WW1 as a response variable in a generalized additive model (GAM) to perform stepwise logistic regression analysis with a binomial error structure, and the logit link function. Our initial model incorporated the long-term average maximum temperatures for May to August [24], phenological or growing season productivity predictor variables, net primary productivity (NPP), season begin date (SBD), and season length (SL) as calculated from Normalized Difference Vegetation Index (NDVI) [25], and potential evapotranspiration (PET) [26] to describe the conditions across our study area. Maximum temperatures in May through August were chosen as they represent temperature extremes during the months that willow warblers arrive, establish breeding territories, breed, and fledge their young in Fennoscandia. Potential evapotranspiration represents the atmospheres ability to remove water through evaporative transpiration and is affected by temperature, precipitation, winds, and solar radiation (which is highly correlated with latitude, *see* Table S2) [27]. Before running the model, we removed predictor variables with high collinearity (>0.7); keeping only one of these variables in the model. We dropped the maximum temperature predictor variable for June, July and August due to their high collinearity with May (*R* = 0.93, *R* = 0.87, and *R* = 0.94, respectively). In addition, we dropped the variables SBD and NPP as they were highly correlated with SL (*R* = 0.92 and *R* = 0.92, respectively). For each model we assessed the significance of each predictor variable by testing the reduction in deviance as measured by the χ^2^ statistic and removed insignificant terms. Finally, to evaluate how well our final model described the conditions across our study area, we used a GAM with the residuals from the final environmental model as the response variable to determine if latitude, longitude, their interaction or altitude explained a significant amount of the residual variance.

To visualize the model predicted northern-allele frequency distribution for the entire region we used the Spatial Analyst ordinary kriging function in ArcMap 10 [28] to create an interpolated continuous grid surface. We then used the resulting interpolated surface to create allele frequency contours in 0.1 increments with the 0.5 isocline representing the center of the cline.

All analyses were performed in R version 2.14 [28].

### Ethics statement

Willow warblers (*Phylloscopus trochilus*) were captured, ringed, bled, and released with permission from Naturvårdsverket and the Swedish Ringing Centre for capturing birds in Sweden (SB - License # 555) and from the Directorate of Nature Management and the Norwegian Ringing Centre in Norway (JTL - License # 159). Blood samples were collected with ethical permission in Sweden from Malmö/Lund djurförsöksetiska nämnd (SB - M 109-02, M 64-05, M 27-08, M 160-11) and permission in Norway explicitly permitted under the ringing permit (JTL - License # 159). Willow warblers are very common and not registered as an endangered or protected species in any country. All samples were collected on lands freely accessible to the public and researchers.

## Results

### Geographic analysis

**Figure 2:**
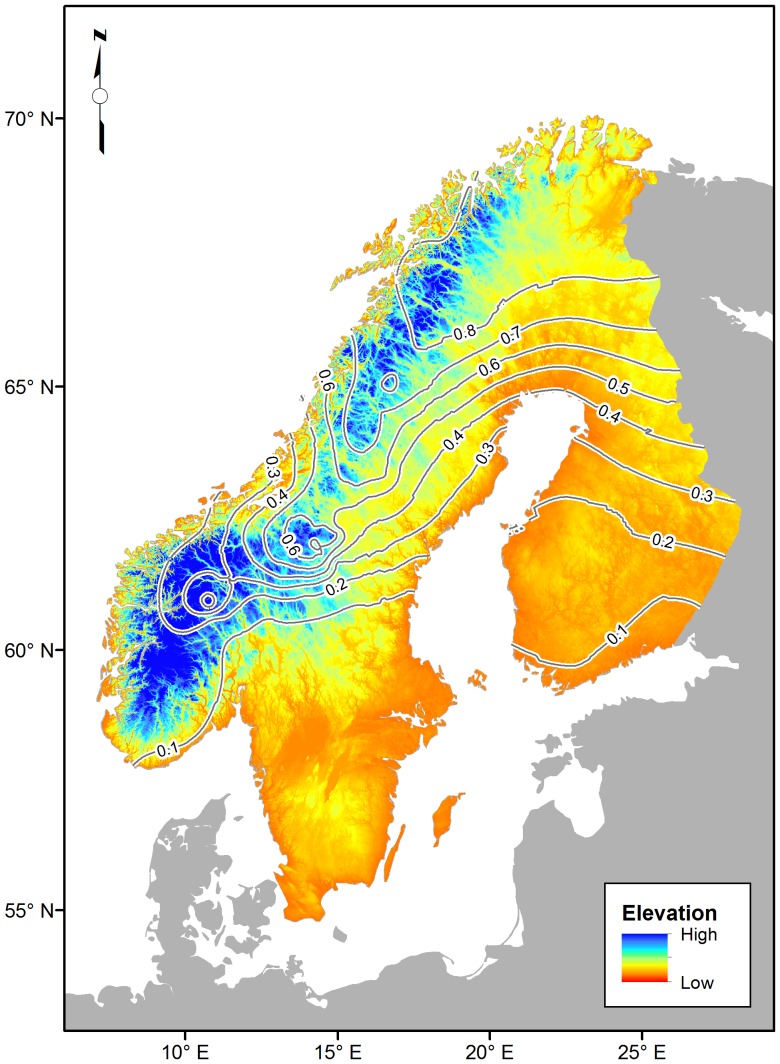
Spatially interpolated predicted 0.1 northern-allele frequency contours for the locus WW1 from the GAM logistic regression geographic model overlaid on elevation background. The 0.5 isocline represents the center of the cline. The background represents a hill-shade digital elevation model (DEM). The map has a 30 arc-second resolutions and is projected using the Swedish RT 90 0 gon Mercator projection.

Geography (latitude, longitude, and their interaction) and altitude were strongly associated with the frequency of the northern-allele of WW1 (R^2^_adj_ = 0.93, Deviance Explained  = 91.7%) across Norway, Sweden, and Finland. The effects of latitude, the interaction between latitude and longitude, and altitude were all significant, while longitude was not significant (Table 1). The frequency for the northern-allele at locus WW1 across Fennoscandia is virtually zero in southernmost Finland and Sweden, and reaches its highest levels (>0.9) in the far north of Fennoscandia (Figure 2). In Finland there is a gradual increase in the northern-allele frequency from 0.1 in the south to 0.9 in the north. Here the cline is shallow with wide contour intervals and oriented north-to-south (Figure 2). In Scandinavia, the cline is more hump-shaped with a lower northern-allele frequency on both the coasts of Norway and Sweden and high allele-frequency in the mountains in-between. In the mountains the cline is steep with narrow contour intervals and oriented north-to-south (Figure 2). Thus, the distribution of the northern-allele is a north-to-south cline across the region with an increasing cline width from west-to-east, as the landscape shifts from mountains to lowlands.

**Table 1:** Parameter estimates for the GAM cline model examining geographical (i.e., latitude, longitude, and their interaction) and altitude variation in the northern-allele frequency for the AFLP marker WW1.

parametric coefficients				
**parameter**	***estimate***	***se***	***z***	***p***
Intercept	−2.21	0.82	−2.67	0.008
Altitude	0.00	0.00	6.80	<0.001
Longitude	0.05	0.05	1.00	0.32
**approximate significance of smooth terms**				
	***est. df***	***fef. df***	***χ^2^***	***p***
Latitude	5.87	9	30.73	<0.001
Latitude*Longitude	12.34	23	32.32	<0.001

Finally, because our sampling efforts contain a large number of sites in the willow warbler contact zone (N = 65; Figure 1) we reran the geographic model to exclude these sites. We did this to emphasize that the patterns are not confounded by the well sampled contact zone neither nor, the differences between the two subspecies. In the model excluding the contact zone, geography (latitude, longitude, and their interaction) and altitude were even more strongly associated with the frequency of the northern-allele of WW1 (R^2^_adj_ = 0.97, Deviance Explained  = 94.5%) across Norway, Sweden, and Finland. The effects of latitude, the interaction between latitude and longitude, and altitude continued to be all significant, while longitude remained not significant (Table S2).

### Environmental analysis

The environmental model demonstrated the significance of climate and growing season variables in predicting the distribution for the northern-allele of WW1 across Fennoscandia (R^2^_adj_ = 0.85, Deviance Explained  = 85.1%). The final model included the significant predictor variables May maximum temperature and potential evapotranspiration (PET) (Table 2). Season length was not significant when evaluated using a χ^2^ test for deviance reduction (*Δ Dev.* = −6.32, *P* = 0.14) and was removed from the model. The final model demonstrates the significance of the maximum temperature in May and PET (*Δ Dev.* = −96.88, *P*<0.0001, *Δ Dev.* = −15.89, *P*<0.04, respectively,) in predicting the frequency distribution of the northern-allele (Figure S1).

**Table 2:** Parameter estimates for the GAM model examining variation in the northern-allele frequency for the AFLP-WW1, where insignificant terms were removed using stepwise regression evaluating the reduction in deviance and significance.

parametric coefficients				
**parameter**	***estimate***	***se***	***m***	***p***
Intercept	−0.69	0.05	−14.17	<0.001
**approximate significance of smooth terms**				
	***est. df***	***fef. df***	***χ^2^***	***p***
Max Temp. May	7.57	8.39	95.15	<0.001
PET	7.35	8.24	13.85	0.10

Final model variables include the mean monthly maximum temperature for May and mean annual potential evapotranspiration (PET).

Using a post hoc test of these results from the environmental model, we used regression to model the residuals against latitude, longitude, their interaction and altitude, and found no significance for any of these geographic variables. We repeated this process using the residuals from the geographic model regressed against May maximum temperature, NPP, and PET, and found no significance for any of these environmental variables.

## Discussion

Our results demonstrate that the northern-allele of the WW1 locus is strongly correlated with both altitude and latitude. Sites dominated by individuals with the northern-allele are characterized by low summer temperatures and a short growing season [29], [30]. This suggests it may be a marker associated with local adaptation for alpine breeding in Fennoscandian willow warblers. The use of our expanded dataset also demonstrated that lowland *acredula* populations in Finland had a significantly higher frequency of southern than northern-alleles, similar to those in coastal Norway where habitats tend to be milder and lower in altitude. Further, our results confirm that the distribution of the two alleles is better explained by our climate model than by the distribution of the subspecies. Lowland coastal breeding *acredula* have a high proportion of the southern allele and mountainous breeding *trochilus* in Southern Norway have high proportion of the northern allele. These patterns suggest that climate is a selective force for WW1 independent of subspecies and their migratory behaviour.

Although the environmental model found climate and growing season conditions as significant predictors of the northern-allele distribution, these variables are highly correlated with both latitude and altitude (Table S1). Therefore, these variables may simply reflect the conditions found in these habitats and serve as a useful proxy for other factors that represent a possible agent of selection. Birds that breed at high latitudes and altitudes often experience greater daily and seasonal climate stochasticity and extremes in climatic conditions, such as temperature, wind, precipitation, and snow cover than those in other habitats [31]–[33]. For example, differences in the timing of summer snow melt can vary by as much as one month between high and low elevation habitats [33]. Further, years of exceptionally harsh conditions and frequent severe storms may make it difficult for potential breeders to acquire food, increase individual thermoregulatory costs, and limit opportunities to breed during the short summer season encountered at these latitudes and altitudes [31]–[33].

At present, we do not know what variables in these mountainous habitats drive the northern-allele to high frequencies or which gene or genes surrounding WW1 that are the targets of selection. Many species of birds show adaptations to living at high altitudes. For example, the bar-headed goose (*Anser indicus*) breeds and migrates at high altitude on the Tibetan Plateau [34] and the Andean goose (*Cheophaga melatoptera*) can reside year-round at over 6000 meters [35], where hypoxic conditions can limit effective transport of blood oxygen, requiring adaptations to respiratory and haemoglobin systems. Because of the relatively low altitudes of the Scandinavian mountains, altitude of our study sites ranges from sea level to 1093 meters, it seems unlikely that WW1 represents adaptions to hypoxic environments, but instead reflects phenological correlates, such as summer temperatures or food conditions in these high latitude regions. We propose two alternative hypotheses for the adaptive value of WW1-linked genes relating to these selective constraints.

As previously discussed, climatic conditions can vary dramatically, daily and seasonally, at high altitudes and latitudes. Birds must be able to modulate their stress response to these conditions in order to successfully breed during the short breeding season [31], [32], [36]. The adrenocortical stress response can suppress breeding behaviour in birds in order to buffer physiological needs essential for survival during harsh weather [31], [36]. Experiments with high altitude and latitude birds, including willow warbler [36], have shown that individuals exposed to stress were better able to modulate the adrenocortical response to stress than others at lower altitudes and latitudes, which makes them resilient to the effects of stress during breeding efforts [31], [36]. Thus, the northern-allele for WW1 may be associated with genetic variation that enables willow warblers to buffer these harsh conditions and maintain a constant reproductive output, which would increase fitness where breeding opportunities are constrained by a short summer season.

Alternatively, selection on genes linked to the northern-allele (WW1) may confer adaptation to a particular diet found at high altitudes and latitudes. Stands of almost pure subalpine birch (*Betula pubescens czerepanovii*) characterize habitats occupied by the “mountain” phenotype (i.e. those with the northern-allele) willow warblers. During the breeding season willow warblers that inhabit subalpine birch communities forage extensively on autumnal moth larvae (*Epirrita autumnata*) which cyclically reach epidemic proportions [37], [38]. In years where these moths reach peak densities, subalpine birch produce defensive secondary-chemical compounds known to be proteinase inhibitors [37]. It is therefore tempting to speculate that the northern-allele for WW1 represents an adaptation to dealing with accumulated secondary chemical compounds in larval autumnal moths.

Although our approach does not allow us to identify the mechanisms leading to positive selection for the WW1 northern-allele, we have identified environmental conditions that explain the distribution of the WW1 alleles. Our results indicate that allelic variation in the genomic region associated with WW1 is positively correlated with clines in the climate. These results open up an avenue for studies of functional genetics to identify the genes underlying the various adaptations to ecological/climatic conditions. Further, this marker provides a genetic tool to study how climate exerts selection on a genomic region in a bird and hence would make an excellent candidate to predict population changes that result from expected future changes in climate.

These results are interesting given the predictions that cold-adapted and mountain populations of a diversity of taxa are particularly vulnerable to extinction due to the rapid climate warming [39]–[41]. In these habitats populations are limited in their ability to disperse upwards to higher altitude areas or northwards and face disproportionately greater risks of extinction in light of significant climate change [40], [41]. Studies of high elevation plants in Europe predict species loss may be as great as 60 percent due to their inability to disperse from these isolated habitats or adapt to warming conditions [40]. In particular, maps of regional plant species vulnerability show almost perfect concordance with the observed northern-allele frequency distribution in Fennoscandian willow warblers [40].

Future work should focus on cold tolerance and food choice experiments to contrast mountain with lowland populations in Fennoscandia, to determine the physiological phenotype associated with the geographical and environmental distribution pattern revealed in this study. Experimental elucidation of physiological differences between genotypes will hopefully lead to the discovery of the direct selective mechanisms linked to these apparent adaptations and the genes associated with this AFLP marker.

## Supporting Information

Figure S1 Spatially interpolated predicted 0.1 northern-allele frequency contours for the locus WW1 from the GAM logistic regression model overlaid on altitude hill shade background. The 0.5 isocline represents the center of the cline. The background represents a hill-shade digital elevation model (DEM). Map has a 30 arc-second resolutions and is projected using the Swedish RT 90 0 gon Mercator projection.
(TIF)

Table S1 The Pearson's correlation coefficients between the predictor variables latitude (lat), longitude (long), altitude (alt), maximum temperature (Max Temp) for May to August, potential evapotranspiration (PET), net primary productivity (NPP), season begin date (SBD), and season length (SL) used in geographical or environmental models.
(DOCX)

Table S2 Parameter estimates for the GAM cline model examining geographical (i.e., latitude, longitude, and their interaction) and altitude variation in the northern-allele frequency for the AFLP marker WW1, excluding sites (N = 65) from the contact zone.
(DOCX)
