# Supplementary figures and images for: Allelic Variation in a Willow Warbler Genomic Region Is Associated with Climate Clines
Source: PLoS One. 2014 May 1;9(5):e95252. doi: 10.1371/journal.pone.0095252 (PMC4006793; doi:10.1371/journal.pone.0095252)

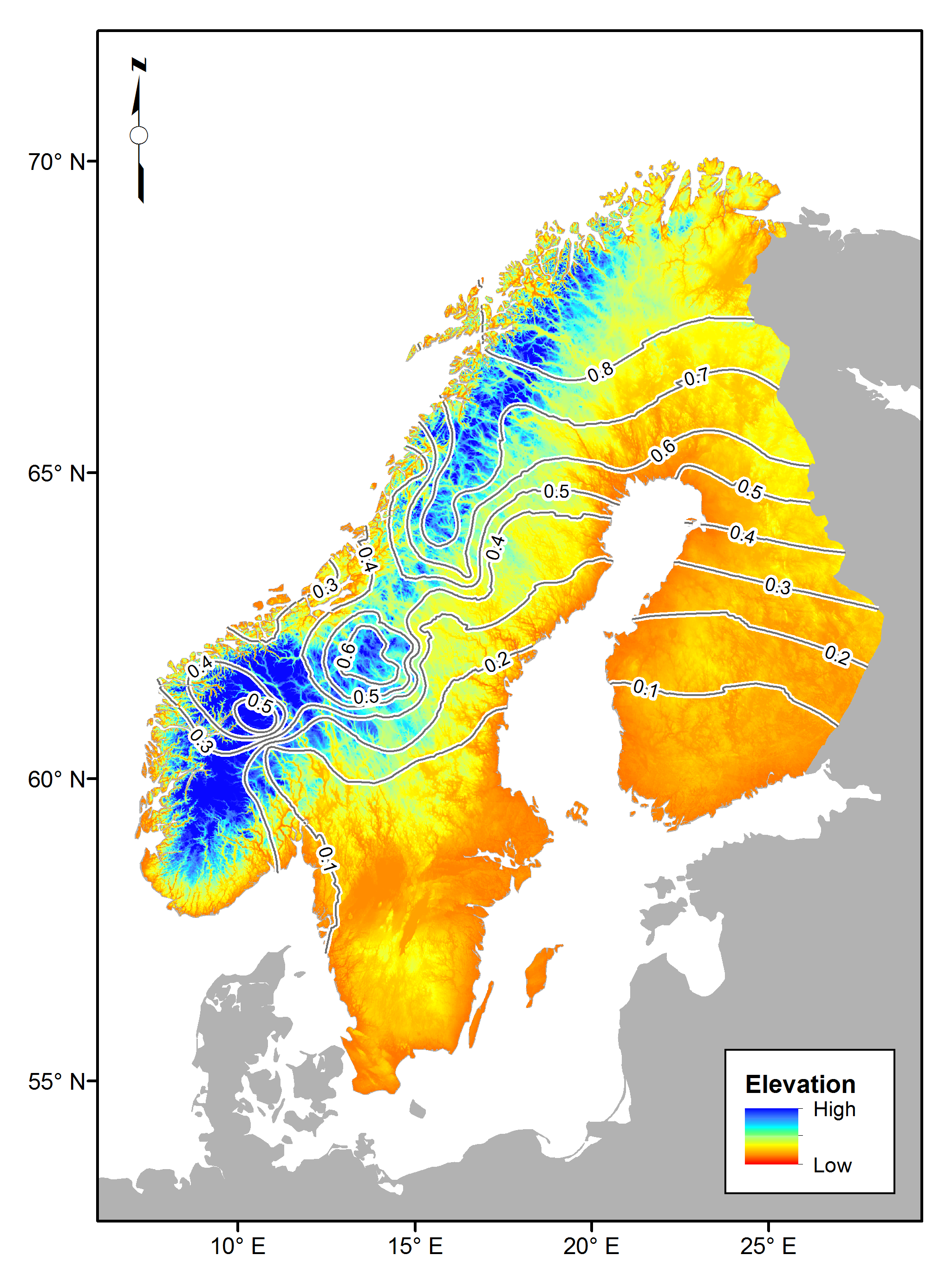

Supplement: Figure S1 — Spatially interpolated predicted 0.1 northern-allele frequency contours for the locus WW1 from the GAM logistic regression model overlaid on altitude hill shade background. The 0.5 isocline represents the center of the cline. The background represents a hill-shade digital elevation model (DEM). Map has a 30 arc-second resolutions and is projected using the Swedish RT 90 0 gon Mercator projection. (TIF) [file pone.0095252.s001.tif]
