# Supplementary material for: Allelic Variation in a Willow Warbler Genomic Region Is Associated with Climate Clines
Source: PLoS One. 2014 May 1;9(5):e95252. doi: 10.1371/journal.pone.0095252 (PMC4006793; doi:10.1371/journal.pone.0095252)
Supplement: Table S1 — The Pearson's correlation coefficients between the predictor variables latitude (lat), longitude (long), altitude (alt), maximum temperature (Max Temp) for May to August, potential evapotranspiration (PET), net primary productivity (NPP), season begin date (SBD), and season length (SL) used in geographical or environmental models. (DOCX) [file pone.0095252.s002.docx]

Table S1

|  |  |  |  | **Temperature Maximum** | | | |  |  |  |  |
| --- | --- | --- | --- | --- | --- | --- | --- | --- | --- | --- | --- |
|  | **Lat** | **Long** | **Alt** | **May** | **June** | **July** | **August** | **PET** | **NPP** | **SBD** | **SL** |
| Lat | 1.00 | 0.56 | 0.05 | -0.83 | -0.67 | -0.56 | -0.69 | -0.79 | -0.46 | 0.22 | -0.43 |
| Long | 0.56 | 1.00 | -0.40 | -0.26 | 0.00 | 0.15 | -0.04 | -0.17 | -0.20 | -0.04 | -0.13 |
| Alt | 0.05 | -0.40 | 1.00 | -0.40 | -0.45 | -0.55 | -0.59 | -0.32 | -0.34 | 0.51 | -0.43 |
| TMax May | -0.83 | -0.26 | -0.40 | 1.00 | 0.93 | 0.87 | 0.94 | 0.96 | 0.69 | -0.51 | 0.67 |
| TMax June | -0.67 | 0.00 | -0.45 | 0.93 | 1.00 | 0.98 | 0.96 | 0.96 | 0.65 | -0.53 | 0.67 |
| TMax July | -0.56 | 0.15 | -0.55 | 0.87 | 0.98 | 1.00 | 0.95 | 0.91 | 0.61 | -0.55 | 0.66 |
| TMax August | -0.69 | -0.04 | -0.59 | 0.94 | 0.96 | 0.95 | 1.00 | 0.93 | 0.64 | -0.55 | 0.67 |
| PET | -0.79 | -0.17 | -0.32 | 0.96 | 0.96 | 0.91 | 0.93 | 1.00 | 0.60 | -0.45 | 0.61 |
| NPP | -0.46 | -0.20 | -0.34 | 0.69 | 0.65 | 0.61 | 0.64 | 0.60 | 1.00 | -0.69 | 0.85 |
| SBD | 0.22 | -0.04 | 0.51 | -0.51 | -0.53 | -0.55 | -0.55 | -0.45 | -0.69 | 1.00 | -0.93 |
| SL | -0.43 | -0.13 | -0.43 | 0.67 | 0.67 | 0.66 | 0.67 | 0.61 | 0.85 | -0.93 | 1.00 |
