# Supplementary material for: Allelic Variation in a Willow Warbler Genomic Region Is Associated with Climate Clines
Source: PLoS One. 2014 May 1;9(5):e95252. doi: 10.1371/journal.pone.0095252 (PMC4006793; doi:10.1371/journal.pone.0095252)
Supplement: Table S2 — Parameter estimates for the GAM cline model examining geographical (i.e., latitude, longitude, and their interaction) and altitude variation in the northern-allele frequency for the AFLP marker WW1, excluding sites (N = 65) from the contact zone. (DOCX) [file pone.0095252.s003.docx]

Table S2

| **parametric coefficients** |  |  |  |  |
| --- | --- | --- | --- | --- |
| **Parameter** | ***estimate*** | ***se*** | ***z*** | ***p*** |
| Intercept | -0.64 | 0.57 | -1.14 | 0.25 |
| Altitude | 0.00 | 0.00 | 4.92 | < 0.001 |
| Longitude | -0.04 | 0.03 | -1.15 | 0.25 |
| **approximate significance of smooth terms** | | |  |  |
|  | ***est. df*** | ***fef. df*** | ***χ^2^*** | ***p*** |
| Latitude | 4.31 | 9 | 251.91 | < 0.001 |
| Latitude*Longitude | 4.54 | 11 | 13.95 | < 0.001 |
